# Risk factors for proliferative vitreoretinopathy after retinal detachment surgery: A systematic review and meta-analysis

**DOI:** 10.1371/journal.pone.0292698

**Published:** 2023-10-30

**Authors:** Jinjin Xiang, Jingjing Fan, Jiahui Wang

**Affiliations:** 1 Department of Ophthalmology, Jiangdu People’s Hospital Affiliated to Medical College of Yangzhou University, Yangzhou, 225200, Jiangsu, China; 2 Department of Ophthalmology, Xian First Hospital, Xi’an, 710002, Shaanxi, China; Taipei Veterans General Hospital | National Yang Ming Chiao Tung University, TAIWAN

## Abstract

**Background:**

To comprehensively investigate risk factors for proliferative vitreoretinopathy (PVR) after retinal detachment (RD) surgery.

**Methods:**

PubMed, Embase, Cochrane Library, and Web of Science were systematically searched until May 22, 2023. Risk factors included demographic and disease-related risk factors. Odds ratios (ORs) and weighted mean differences (WMDs) were used as the effect sizes, and shown with 95% confidence intervals (CIs). Sensitivity analysis was conducted. The protocol was registered with PROSPERO (CRD42022378652).

**Results:**

Twenty-two studies of 13,875 subjects were included in this systematic review and meta-analysis. Increased age was associated with a higher risk of postoperative PVR (pooled WMD = 3.98, 95%CI: 0.21, 7.75, *P* = 0.038). Smokers had a higher risk of postoperative PVR than non-smokers (pooled OR = 5.07, 95%CI: 2.21–11.61, *P*<0.001). Presence of preoperative PVR was associated with a greater risk of postoperative PVR (pooled OR = 22.28, 95%CI: 2.54, 195.31, *P* = 0.005). Presence of vitreous hemorrhage was associated with a greater risk of postoperative PVR (pooled OR = 4.12, 95%CI: 1.62, 10.50, *P* = 0.003). Individuals with aphakia or pseudophakia had an increased risk of postoperative PVR in contrast to those without (pooled OR = 1.41, 95%CI: 1.02, 1.95, *P* = 0.040). The risk of postoperative PVR was higher among patients with macula off versus those with macula on (pooled OR = 1.85, 95%CI: 1.24, 2.74, *P* = 0.002). Extent of RD in patients with postoperative PVR was larger than that in patients without (pooled WMD = 0.31, 95%CI: 0.02, 0.59, *P* = 0.036). Patients with postoperative PVR had longer duration of RD symptoms than those without (pooled WMD = 10.36, 95%CI: 2.29, 18.43, *P* = 0.012).

**Conclusion:**

Age, smoking, preoperative PVR, vitreous hemorrhage, aphakia or pseudophakia, macula off, extent of RD, and duration of RD symptoms were risk factors for postoperative PVR in patients undergoing RD surgery, which may help better identify high-risk patients, and provide timely interventions.

## Background

The success of retinal detachment (RD) surgery is dependent on the absence or control of proliferative vitreoretinopathy (PVR) [1]. PVR, one of the most important complications following vitreoretinal surgery, is a fibrotic eye disease with an incidence of 5–11%, and characterized by migration and proliferation of cells following a break in the retina or trauma, leading to formation of membranes in the periretinal area, followed by contraction of the cellular membranes and traction on the retina that causes RD [2–4]. For improvements in the surgical success rate and prevention of this complication, it is necessary to understand the risk factors for PVR in patients after RD surgery.

Several studies have been performed to explore the risk factors for PVR after RD surgery, but there are still inconsistent results. In the study of Xu et al. [5], macular involvement and cigarette smoking are factors predictive of PVR formation after uncomplicated primary RD repair. Bonnet et al. [6] illustrated that preoperative grade-B PVR and preoperative vitreous hemorrhage were associated with a significantly higher incidence of postoperative PVR. A history of uveitis related to an increased risk of PVR following RD surgery, according to another study [7]. In cases of RD with pre-existing PVR, there is a significant risk of progression to further advanced PVR [8–10]. Smoking was reported by Eliott et al. [11] to be a risk factor for PVR after the repair of RD associated with open-globe trauma. Another study showed that patients >70 years and with intraocular pressure lower than 14 mmHg had a higher risk of PVR after RD surgery [12]. At present, only a systematic review protocol of Chaudhary et al. [10] is available, and no study comprehensively evaluates the risk factors for PVR after RD surgery.

This study intended to comprehensively probe into the risk factors for PVR after RD surgery via a systematic review and meta-analysis, and focused on demographic risk factors and disease-related risk factors.

## Methods

### Search strategy

PubMed, Embase, Cochrane Library, and Web of Science were systematically searched until May 22, 2023 by two independent reviews (JJ Fan, JH Wang). Disagreements were resolved through discussion. English search terms included “Retinal Detachment” OR “Detachment, Retinal” OR “Detachments, Retinal” OR “Retinal Detachments” OR “Retinal Pigment Epithelial Detachment” AND “Vitreoretinopathy, Proliferative” OR “Proliferative Vitreoretinopathies” OR “Vitreoretinopathies, Proliferative” OR “Vitreoretinopathy Neovascular Inflammatory” OR “Inflammatories, Vitreoretinopathy Neovascular” OR “Inflammatory” OR “Vitreoretinopathy Neovascular” OR “Neovascular Inflammatories, Vitreoretinopathy” OR “Neovascular Inflammatory, Vitreoretinopathy” OR “Vitreoretinopathy Neovascular Inflammatories” OR “Proliferative Vitreoretinopathy”. Review studies were hand-searched to identify additional articles. This systematic review and meta-analysis followed the Preferred Reporting Items for Systematic Reviews and Meta-Analyses (PRISMA) guidelines. The protocol was registered with PROSPERO (CRD42022378652).

### Eligibility criteria

Inclusion criteria were: (1) studies on patients undergoing RD surgery; (2) studies reporting whether patients had PVR after RD surgery; (3) studies reporting at least one of the following: demographic risk factors and disease-related risk factors; (4) cohort studies and case-control studies; (5) studies in English; (6) the latest studies when the study population was repeated.

Exclusion criteria were: (1) studies with animal experiments; (2) studies unrelated to the research topic, such as studies on drug testing, etc.; (3) studies for which data are incomplete or data cannot be extracted; (4) conference reports, dissertations, case reports, meta-analyses, reviews.

### Data extraction and quality assessment

Extracted data included first author, year of publication, country, study period, study design, sample size, preoperative PVR, sex ratio (male/female), age (years), follow-up time (months), disease type, type of surgery, quality assessment, and risk factor. The quality of the included case-control and cohort studies was evaluated using the modified Newcastle-Ottawa scale (NOS). The scale had a total score of 9, with 0–3 as low quality, 4–6 as medium quality, and 7–9 as high quality [13].

### Statistical analysis

All studies were statistically analyzed using Stata 15.1 (Stata Corporation, College Station, TX, USA). For enumeration data, odds ratios (ORs) and hazard ratios (HRs) were used as the effect sizes, and for measurement data, weighted mean differences (WMDs) acted as the effect size, with 95% confidence intervals (CIs) calculated. The heterogeneity test was carried out for each index. If the heterogeneity statistic I^2^≥50%, the random-effects model was used for analysis; otherwise, the fixed-effects model was used for analysis. Sensitivity analysis was carried out for all models. *P*<0.05 was considered to be statistically significant.

## Results

### Characteristics of the included studies

**Fig 1:**
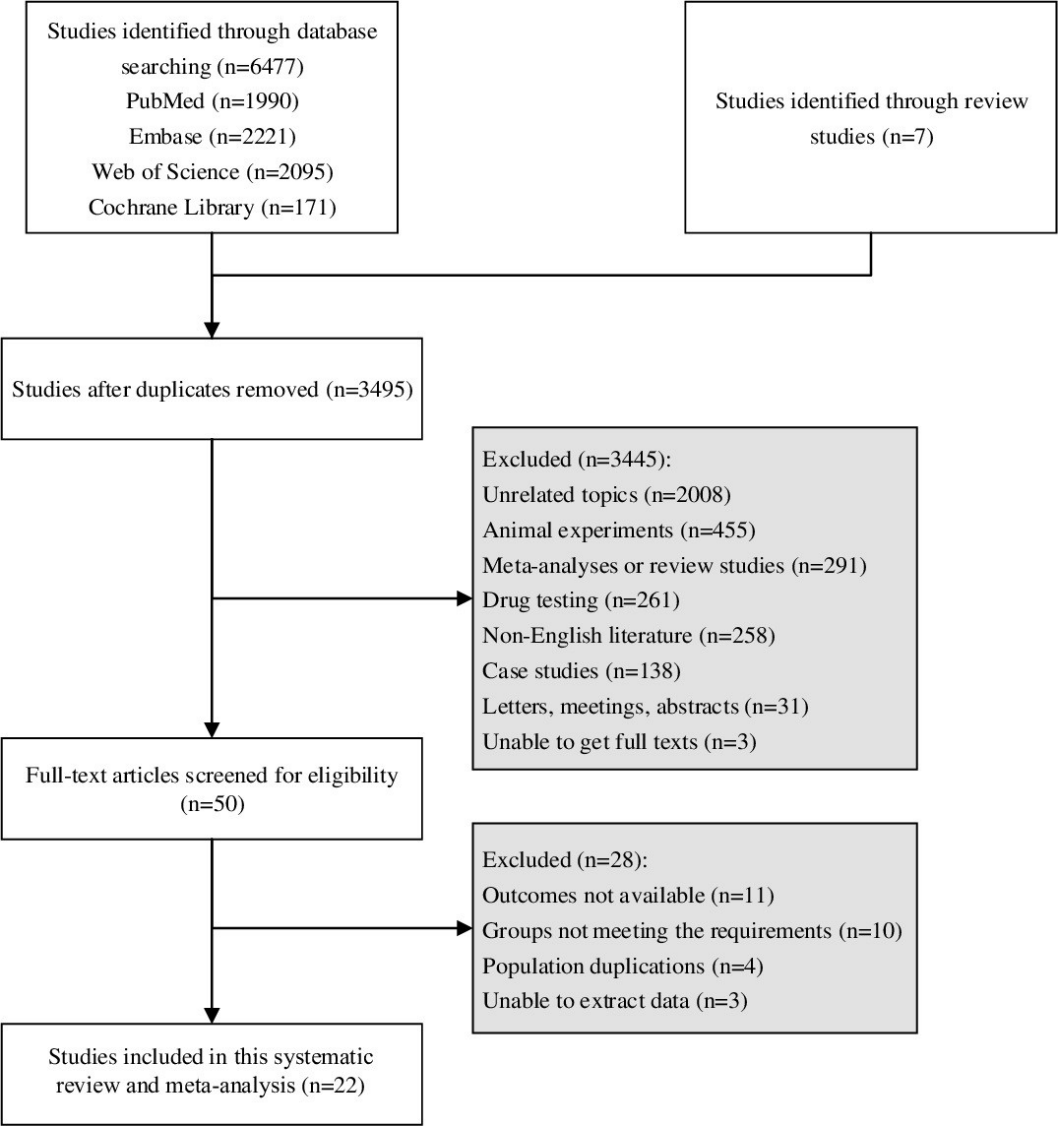
Flow chart for study screening.

A total of 6,477 studies were retrieved from the four databases, and 7 additional articles were identified from review studies. After deduplication, 3,495 were left. Subsequently, titles and abstracts and then full texts were subject to screening according to the eligibility criteria. Finally, 22 studies [5,7,11,12,14–30] of 13,875 subjects were included, published between 1984 and 2023. Fig 1 shows a flow chart of study selection. Three studies did not involve patients with preoperative PVR, some patients in 16 studies had preoperative PVR, and 3 studies did not report preoperative PVR. Of the included studies, 7 were cohort studies and 15 were case-control studies. All the studies were evaluated to have medium quality. Basic characteristics of the included studies are illustrated in Table 1.

**Table 1:** Basic characteristics of the included studies.

Author	Year	Country	Study period	Study design	Sample size	Preoperative PVR	Sex ratio (M/F)	Age (years)	FU time (months)	Disease type	Type of surgery	QA	Risk factor
Gao	2023	China	2016–2020	Case control	741	Mixed	423/318	≤40 155, 41–50 126, 51–60 215, 61–70 197, >70 48	≥6	Primary RRD	Vitrectomy	6	Sex, age, preoperative PVR
Kasetty	2023	USA	2010–2020	Retrospective cohort	62	-	25/37	15–24 10, 25–34 14, 35–45 38	33	RRD	PPV	4	Age
Loiudice	2023	Italy	2018–2020	Case control	85	Mixed	58/27	61.73±11.24	3	Primary RRD	PPV	4	Age, aphakia or pseudophakia, macula off
Ferrara	2022	UK	2011–2019	Retrospective cohort	8133	Mixed	5182/2951	59.13 (16–100)	≥2	Primary RRD	Vitrectomy 7064, SB 769, vitrectomy+SB 112, pneumatic retinopexy 188	4	Age
Kocak	2022	Turkey	2017–2021	Case control	150	No	97/53	57.86±12.40	3	Primary RRD	PPV	5	Sex, age, aphakia or pseudophakia, macula off, extent of RD
Moussa	2022	UK	2017–2021	Retrospective cohort	259	Mixed	187/72	61 (49–71)*	6	Primary RRD	PPV	4	Sex, age, preoperative PVR, macula off
Patel	2021	USA	2015–2018	Case control	69	Mixed	51/18	42.9±15.5	≥3	Primary RRD	SB	6	Sex, age, smoking, preoperative PVR, history of uveitis, surgical history, VH, aphakia or pseudophakia, macula off, IOP, retinal breaks, duration of RD symptoms
Zandi	2021	Switzerland	-	Case control	65	No	40/25	61.3±13.4	6	Primary RRD	PPV	6	Sex, age, aphakia or pseudophakia, macula off, retinal breaks
Antaki	2020	Canada	2012–2019	Case control	506	Mixed	341/165	18–95	52 (3–105)	RRD	PPV	5	Sex, age, preoperative PVR, history of uveitis, surgical history, VH, aphakia or pseudophakia, macula off, IOP, retinal breaks, duration of RD symptoms
Slingsby	2020	USA	2011–2017	Retrospective cohort	523	-	-	3–90	8.5	Primary RRD	-	5	History of uveitis
Xu	2019	USA	2014–2015	Case control	74	No	45/29	61.9±12.0	12.4±5.3	Primary RD	SB 7, PPV 32, SB+PPV 35	6	Sex, age, smoking, VH, aphakia or pseudophakia, IOP, retinal breaks, duration of RD symptoms
Mulder	2018	the Netherlands	2014	Case control	195	Mixed	127/68	59.2±11.0	≥6	Primary RRD	SB 70, PPV 125	4	Sex, age, aphakia or pseudophakia
Conart	2017	France	2013	Case control	100	Mixed	55/45	59.49±12.93	≥6	Primary RRD	SB 20, PPV 80	5	Age, VH, aphakia or pseudophakia, macula off, extent of RD
Eliott	2017	USA	1999–2011	Case control	138	Mixed	107/31	42 ± 22	≥6	RD	SB 3, PPV 70, SB+PPV 65	4	Smoking, preoperative PVR
Ricker	2012	the Netherlands	2001–2008	Case control	75	Mixed	45/20	43–79	3–80	Primary RRD	SB	5	Sex, VH, aphakia or pseudophakia, macula off
Pastor	2005	Spain	1996–2001	Case control	335	Mixed	214/121	54.5 (13–91)	3	RD	SB 169, vitrectomy 166	5	Surgical history, aphakia or pseudophakia
Kon	1999	UK	1995–1996	Prospective cohort	146	Mixed	94/42	59.0 (16–86)	8.3	RRD	PPV	5	Preoperative PVR
Capeans	1998	Spain	-	Case control	65	Mixed	28/37	56.8	6.6 (3–15)	RRD	SB 63, pneumatic retinopexy 5	6	Sex, aphakia or pseudophakia, macula off
Duquesne	1996	France	1984–1993	Prospective cohort	390	Mixed	244/146	50 (4–85)	12	Primary RRD	SB 397, vitrectomy 144	4	Preoperative PVR, VH
Girard	1994	France	1985–1992	Case control	1020	Mixed	-	-	17.1±12.8	RRD	SB 958, vitrectomy 176	4	Preoperative PVR, history of uveitis
Cowley	1989	USA	1978–1984	Case control	390	Mixed	-	58.3	-	RRD	Vitrectomy 54	6	Preoperative PVR, surgical history, VH, aphakia or pseudophakia, macula off
Bonnet	1984	France	-	Prospective cohort	354	-	-	-	≥3	RRD	Release of subretinal fluid 76, intravitreal gas injection 53, SB+ vitrectomy 20	6	Surgical history

* Median (interquartile range).

PVR, proliferative vitreoretinopathy; M/F, male/female; FU, follow-up; QA, quality assessment; IOP, intraocular pressure; RD, retinal detachment; RRD, rhegmatogenous retinal detachment; SB, scleral buckling; PPV, pars plana vitrectomy; VH, vitreous hemorrhage.

### Demographic risk factors

**Fig 2:**
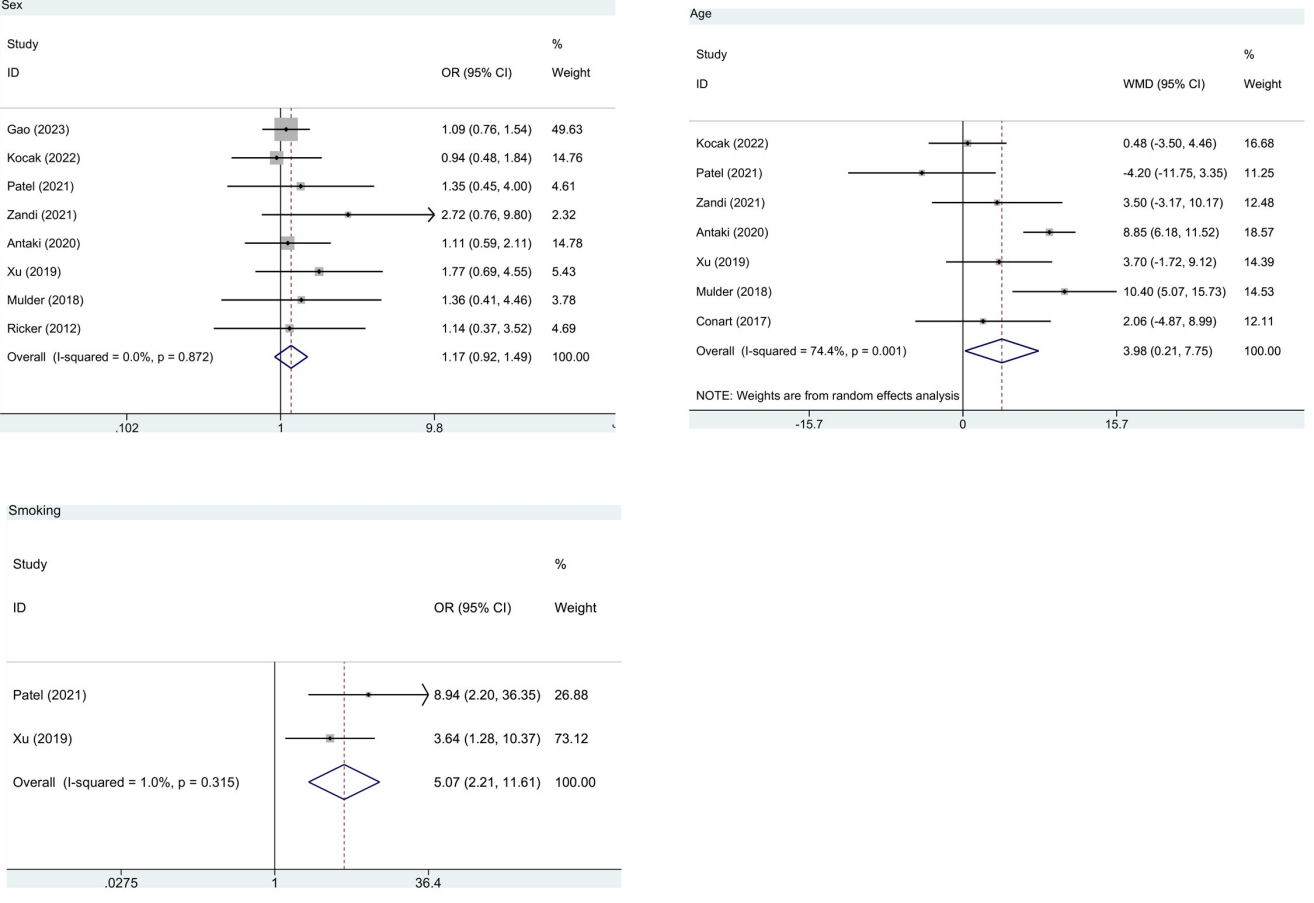
Forest plot for demographic risk factors in patients without PVR before RD surgery. (a) sex; (b) age; (c) smoking. PVR, proliferative vitreoretinopathy; RD, retinal detachment; WMD, weighted mean difference; OR, odds ratio; CI, confidence interval.

**Sex.** Eight studies of 1,875 patients reported the association between sex and the risk of postoperative PVR. No significant difference was observed in the risk of postoperative PVR between male and female patients (pooled OR = 1.17, 95%CI: 0.92, 1.49, *P* = 0.210, I^2^ = 0.0%) (Fig 2A and Table 2).

**Table 2:** Risk factors for PVR after RD surgery.

Risk factor	Pooled OR/WMD (95%CI)	*P*	I²
**Demographic risk factor**			
Sex	1.17 (0.92, 1.49)	0.210	0.0
Age	3.98 (0.21, 7.75)	0.038	74.4
Smoking	5.07 (2.21, 11.61)	<0.001	1.0
**Disease-related risk factor**			
Preoperative PVR	22.28 (2.54, 195.31)	0.005	87.8
History of uveitis	3.68 (0.81, 16.64)	0.091	55.5
Surgical history	1.17 (0.54, 2.53)	0.696	0.0
Vitreous hemorrhage	4.12 (1.62, 10.50)	0.003	52.9
Aphakia or pseudophakia	1.41 (1.02, 1.94)	0.040	0.0
Macula off	1.85 (1.24, 2.74)	0.002	47.2
Intraocular pressure	-0.97 (-3.36, 1.42)	0.427	84.0
Extent of RD	0.31 (0.02, 0.59)	0.036	0.0
Retinal breaks	0.09 (-0.19, 0.38)	0.523	0.0
Duration of RD symptoms	10.36 (2.29, 18.43)	0.012	40.7

PVR, proliferative vitreoretinopathy; RD, retinal detachment; WMD, weighted mean difference; OR, odds ratio; CI, confidence interval.

Moussa et al. showed that sex is not a risk factor for postoperative PVR (OR = 0.60, 95%CI: 0.26–1.39, *P* = 0.234). According to the study of Capeans et al., there is no gender difference between the postoperative PVR group and the postoperative non-PVR group, with a male proportion of 50% vs 42%, respectively.

*Age*. Age was assessed in 7 studies with 1,155 patients. The combined result exhibited that increased age was associated with a significantly higher risk of postoperative PVR (pooled WMD = 3.98, 95%CI: 0.21, 7.75, *P* = 0.038, I^2^ = 74.4%) (Fig 2B and Table 2).

Ferrara et al. found that elderly patients showed more severe PVR (grade C and above), especially after 60 years old. Kasetty et al. illustrated a higher incidence of PVR in young patients. Gao et al., Loiudice et al. and Moussa et al. reported that age is not a risk factor for postoperative PVR.

#### Smoking

As regards smoking, pooled analysis of 2 studies with 143 patients showed that smokers had a significantly higher risk of postoperative PVR than non-smokers (pooled OR = 5.07, 95%CI: 2.21–11.61, *P*<0.001, I^2^ = 1.0%) (Fig 2C and Table 2).

Eliott et al. reported that smoking was a risk factor for redetaching due to postoperative PVR (HR = 1.89, 95%CI: 1.13–3.15, *P* = 0.01).

### Disease-related risk factors

#### Preoperative PVR

**Fig 3:**
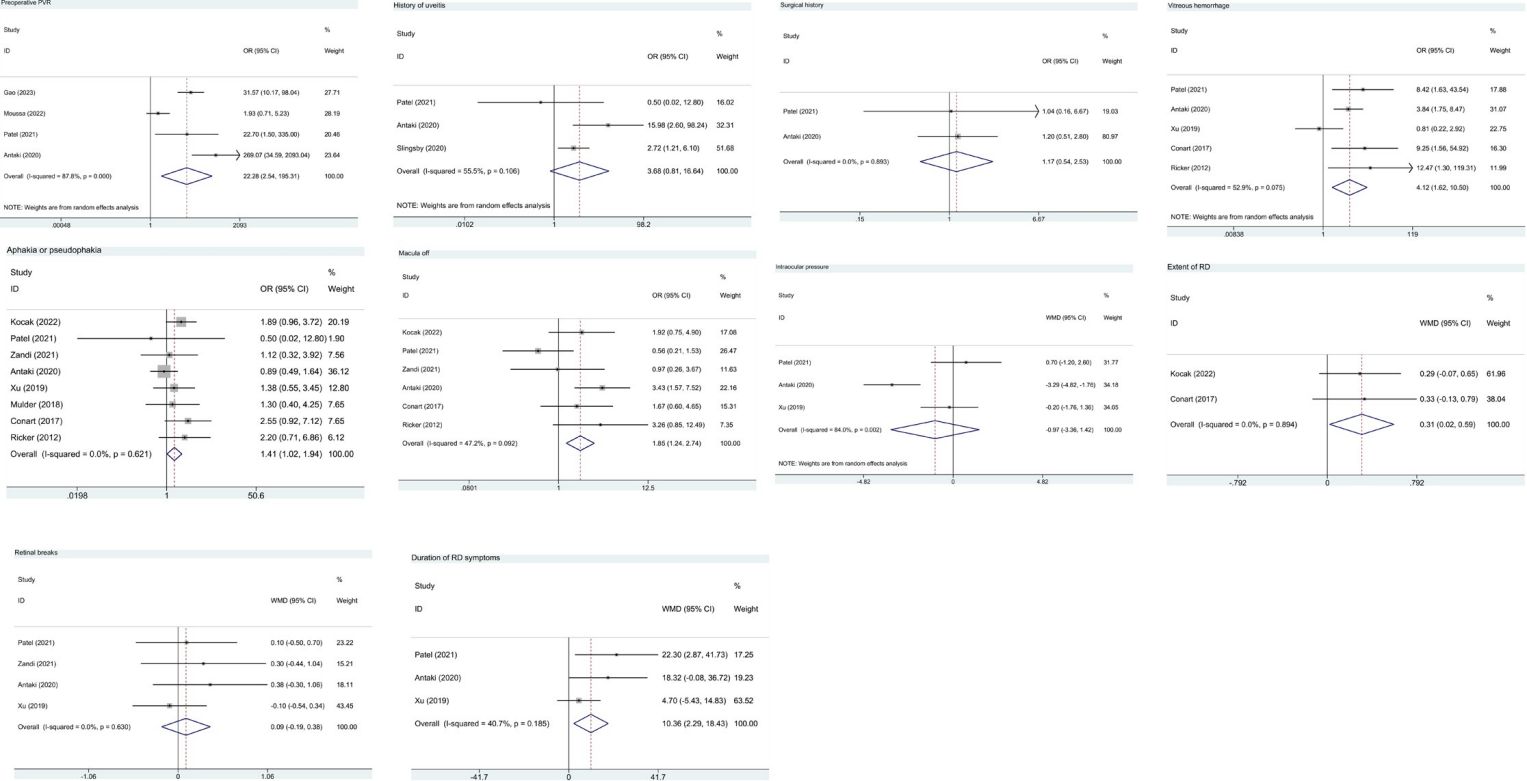
Forest plot for disease-related risk factors in patients without PVR before RD surgery. (a) preoperative PVR; (b) history of uveitis; (c) surgical history; (d) vitreous hemorrhage; (e) aphakia or pseudophakia; (f) macula off; (g) intraocular pressure; (h) extent of RD; (i) retinal breaks; (j) duration of RD symptoms. PVR, proliferative vitreoretinopathy; RD, retinal detachment; WMD, weighted mean difference; OR, odds ratio; CI, confidence interval.

Based on 4 studies of 1,575 patients, presence of preoperative PVR was associated with a significantly greater risk of postoperative PVR (pooled OR = 22.28, 95%CI: 2.54, 195.31, *P* = 0.005, I^2^ = 87.8%) (Fig 3A and Table 2).

The studies of Eliott et al. (HR = 1.75, 95%CI: 1.10, 2.90, *P* = 0.02), Kon et al. (*P*<0.05), Duquesne et al. (preoperative PVR grade B: OR = 8.585, *P* = 0.051; preoperative PVR grade C-D: OR = 50.300, *P*<0.05), Girard et al. (preoperative PVR grade A: OR = 3.690, *P*<0.05; preoperative PVR grade B: OR = 1.491, *P* = 0.047), and Cowley et al. (53.8% vs 11.1%, *P*<0.01) demonstrated that preoperative PVR was a risk factor for redetaching due to postoperative PVR.

#### History of uveitis

The data on a history of uveitis were provided by 3 studies with 1,098 patients. The overall result showed no significant difference in the risk of postoperative PVR between patients with and without a history of uveitis (pooled OR = 3.68, 95%CI: 0.81, 16.64, *P* = 0.091, I^2^ = 55.5%) (Fig 3B and Table 2).

Girard et al. demonstrated that a history of uveitis was associated with a greater risk of postoperative PVR (OR = 1.656, *P* = 0.044).

#### Surgical history

Two studies on 575 patients estimated the risk of postoperative PVR in patients with a history of ophthalmic surgery. A history of ophthalmic surgery was not associated with a greater risk of postoperative PVR (pooled OR = 1.17, 95%CI: 0.54, 2.53, *P* = 0.696, I^2^ = 0.0%) (Fig 3C and Table 2).

Pastor et al. (OR = 1.55, 95% CI: 1.14, 9.22), Cowley et al. (*P*<0.01), and Bonnet et al. (*P*<0.05) showed that previous ophthalmic surgery was a risk factor for postoperative PVR.

#### Vitreous hemorrhage

Vitreous hemorrhage was reported in 5 studies with 820 subjects. Presence of vitreous hemorrhage was associated with a significantly greater risk of postoperative PVR (pooled OR = 4.12, 95%CI: 1.62, 10.50, *P* = 0.003, I^2^ = 52.9%) (Fig 3D and Table 2).

The study of Duquesne et al. showed that 11.3% of patients with preoperative vitreous hemorrhage and 11.8% of patients without preoperative vitreous hemorrhage developed PVR after surgery, respectively (*P* = 0.90). Cowley et al. found that severe vitreous hemorrhage was significantly more frequent in the PVR group.

#### Aphakia or pseudophakia

Eight studies of 1,230 patients demonstrated that individuals with aphakia or pseudophakia had a significantly increased risk of postoperative PVR in contrast to those without (pooled OR = 1.41, 95%CI: 1.02, 1.94, *P* = 0.040, I^2^ = 0.0%) (Fig 3E and Table 2).

Pastor et al. illustrated that aphakic or pseudophaki elevated the risk of postoperative PVR (OR = 3.33, 95%CI: 1.54, 7.22). Loiudice et al. found that lens status was not a predictive factor for the development of postoperative PVR (OR = 0.494, 95%CI: 0.035, 7.064). Capeans et al. (10% vs 7%) and Cowley et al. (47.6% vs 42.6%) showed no difference in aphakic or pseudophaki between the postoperative PVR and non-PVR groups.

#### Macula off

Macula off was assessed in 6 studies with 961 patients. The risk of postoperative PVR was significantly higher among patients with macula off versus those with macula on (pooled OR = 1.85, 95%CI: 1.24, 2.74, *P* = 0.002, I^2^ = 47.2%) (Fig 3F and Table 2).

According to Cowley et al., patients with macula off accounted for 84.6% in the postoperative PVR group and 64.6% in the non-PVR group (*P* = 0.002), indicating that macula off is a risk factor for PVR after RD surgery. Loiudice et al. (OR = 2.13, 95%CI: 0.094, 48.33, *P* = 0.634), and Moussa et al. (OR = 0.52, 95%CI: 0.15, 1.82, *P* = 0.309) showed that macula status was not a risk factor for postoperative PVR. Capeans et al. found no difference in macula off between the postoperative PVR and non-PVR groups (30% vs 41%).

#### Intraocular pressure

Data on intraocular pressure was available in 3 studies of 649 patients. The pooled result indicated no significant association between intraocular pressure and the risk of postoperative PVR (pooled WMD = -0.97, 95%CI: -3.36, 1.42, *P* = 0.427, I^2^ = 84.0%) (Fig 3G and Table 2).

#### Extent of RD

Two studies with 246 patients provided information on extent of RD. Extent of RD in patients with postoperative PVR was significantly larger than that in patients without postoperative PVR (pooled WMD = 0.31, 95%CI: 0.02, 0.59, *P* = 0.036, I^2^ = 0.0%) (Fig 3H and Table 2).

Loiudice et al. showed that extent of RD was not a risk factor for postoperative PVR (OR = 0.30, 95%CI: 0.04, 0. 2.26, *P* = 0.242).

#### Retinal breaks

Retinal breaks were reported in 4 studies of 714 patients. No significant difference was found in retinal breaks between patients with and without postoperative PVR (pooled WMD = 0.09, 95%CI: -0.19, 0.38, *P* = 0.523, I^2^ = 0.0%) (Fig 3I and Table 2).

Loiudice et al. demonstrated that the number of retinal breaks was not associated with PVR after RD surgery (OR = 0.64, 95%CI: 0.52, 2.86, *P* = 0.643).

#### Duration of RD symptoms

Three studies with 649 patients evaluated duration of RD symptoms. The overall results showed that patients with postoperative PVR had significantly longer duration of RD symptoms than those without postoperative PVR (pooled WMD = 10.36, 95%CI: 2.29, 18.43, *P* = 0.012, I^2^ = 40.7%) (Fig 3J and Table 2).

### Sensitivity analysis

Sensitivity analysis was conducted by deleting a study at a time and synthetically assessing the remaining studies. It was found that the pooled results were not affected by one-study deletion, suggesting that the findings of this meta-analysis were stable and robust.

## Discussion

This review and meta-analysis first comprehensively evaluated the risk factors for PVR after RD surgery from the perspectives of demographic and disease-related risk factors. It was revealed that age, smoking, preoperative PVR, vitreous hemorrhage, aphakia or pseudophakia, macula off, extent of RD, and duration of RD symptoms were risk factors for postoperative PVR in patients undergoing RD surgery. Combined assessment of demographic and disease-related risk factors may aid in better prediction of the PVR risk following RD surgery. Awareness of these factors in the development of PVR may allow for early lifestyle interventions and careful surgical planning to minimize the risk.

As regards demographic risk factors, older age was associated with a higher risk of PVR after RD surgery. Apart from the included studies that supported the above statement [12,20,26], no other studies are found to support the statement. Future evidence is needed to confirm this finding. A possible explanation for this finding is that increased signaling of pro-inflammatory cytokines in patients with older age can promote the progression of PVR [31,32]. The lack of research on the underlying mechanism necessitates future studies. We also found that smoking was predictive of postoperative PVR formation. Cigarette smoking may influence the integrity of the blood-retinal barrier since the breakdown of the blood-retinal barrier was considered to be related to the formation of PVR [33]. Afterwards, serum components (such as fibronectin and platelet-derived growth factor) invaded the vitreous and accelerated the migration and proliferation of retinal pigment epithelial cells released into the vitreous after RD. Additionally, smoking was correlated with the development of uveitis and retinal neovascularization [34,35]. Wang et al. [36] indicated that the increase of pro-inflammatory cytokine signaling could play an essential role in the development of PVR in smokers. More investigations are needed to clarify the role of smoking in the risk of PVR after RD surgery. Although smoking was reported to be a risk factor for eye diseases [37], a small number of smokers (about 10–13%) realized that smoking can cause visual impairment [38]. In optometric practice, nearly 45% of optometrists did not evaluate patients’ smoking status at the initial visit and 8% of the optometrists never updated patients’ smoking status [39]. Besides, 2.3% of optometrists never explained the adverse effect of smoking on eye health to patients [39]. Thus, it is very important for vitreoretinal subspecialists, comprehensive ophthalmologists, and optometrists who provide primary eye care to highlight the negative impact of smoking. Eye care professionals should provide smoking cessation programs for patients undergoing RD surgery. Quitting smoking after diagnosis of RD should be advocated as a potential timely intervention for this public health concern.

With respect to disease-related risk factors, patients with preoperative PVR, vitreous hemorrhage, aphakia or pseudophakia, macula off, large extent of RD, and long duration of RD symptoms had an elevated risk of postoperative PVR. Scare evidence has been provided on the role of preoperative PVR in such a setting. Bonnet et al. [6] illustrated that preoperative PVR and vitreous hemorrhage were correlated with a significantly higher incidence of postoperative PVR. According to a prior review, individuals with vitreous hemorrhage had high odds of many diseases, including PVR [40]. We assume that the association between vitreous hemorrhage and postoperative PVR is attributed to high levels of cytokines. Preoperative aqueous flare values may increase as extent of RD becomes large [41], and the flare values may predict PVR re-detachment [17], which may contribute to the association between extent of RD and the risk of postoperative PVR. As regards the relationship between duration of RD symptoms and postoperative PVR, we speculated that long duration of RD symptoms provides time for retinal pigment epithelium cells to migrate, proliferate and generate extracellular matrix, resulting in contracted membrane on the anterior or lower surface of the retina, fixed retinal folds and retinal traction [42]. Concerning the associations of aphakia or pseudophakia and macula off with the risk of PVR following RD surgery, more investigations are warranted to confirm and elaborate on these associations.

This work may act as a reference for vitreoretinal surgeons, and the identified evidence may help the standardization of clinical practice, with more effective management bringing improved outcomes for patients following RD surgery. Given the risk factors, patients should pay attention to and practice smoking cessation and eye care to prevent and lower the risk of postoperative PVR. Targeted screening strategies and interventions could be developed for individuals at a high risk of PVR after RD surgery. Some limitations of this study should be noted. First, the results of combined analysis may be unstable due to the existence of heterogeneity. Second, confounding factors were not taken into account when evaluating these risk factors. Third, the study only included English literature, which may cause language bias. Besides, of 22 included studies, 9 studies [5,9,11,12,15,17,19,20,22] reported a mixture of vitrectomy and scleral buckling (SB) (and, in 2 studies [15,20], other types of surgery), 1 study [16] reported SB and pneumatic retinopexy, and 1 study [7] did not report the type of surgery. Subgroup analysis could not be performed based on the type of surgery (vitrectomy, SB). The aggressiveness and progression of PVR in post vitrectomy and post SB can be different. Pars plana vitrectomy (PPV) plus SB was associated with a higher risk of PVR re-detachment than PPV alone [11]. Additionally, the surgical method may vary among patients with different demographic characteristics. For example, 73% older patients with rhegmatogenous retinal detachment underwent PPV [43]. Thus, pooled evaluation of PVR in all PPV/SB cases may increase the heterogeneity of this study, which further affect the stability of the results. For another, the results of this pooled evaluation may be applicable to patients undergoing either vitrectomy or SB. Future research is needed for verification regarding heterogeneity and applicability. Furthermore, since most studies reported admixed surgical population (some PPV, some SB in each study) and original data among these studies could not be obtained, separate analysis regarding only cases undergoing micro-incision vitrectomy surgery (MIVS) seems not feasible, which requires more explorations.

## Conclusion

Age, smoking, preoperative PVR, vitreous hemorrhage, aphakia or pseudophakia, macula off, extent of RD, and duration of RD symptoms were risk factors for PVR following RD surgery. Future studies are warranted to support our findings.

## Supporting information

S1 Checklist PRISMA 2020 checklist. (DOCX)
